# Cardiovascular protein profiling in patients with first-episode psychosis

**DOI:** 10.1038/s41537-025-00633-x

**Published:** 2025-06-14

**Authors:** Anna Malmqvist, Feride Eren, Lilly Schwieler, Funda Orhan, Helena Fatouros-Bergman, Lena Flyckt, Fredrik Piehl, Simon Cervenka, Magnus Bäck, Carl M. Sellgren, Göran Engberg, Sophie Erhardt

**Affiliations:** 1 Department of Physiology and Pharmacology, Karolinska Institutet, Stockholm, Sweden; 2 Centre for Psychiatry Research, Department of Clinical Neuroscience, Karolinska Institutet & Stockholm Health Care Services, Region Stockholm, Sweden; 3 Neuroimmunology Unit, Department of Clinical Neuroscience, Karolinska Institutet, Stockholm, Sweden; 4 Center for Molecular Medicine, Karolinska Institutet, Karolinska University Hospital, Stockholm, Sweden; 5 Division of Neurology, Karolinska University Hospital, Stockholm, Sweden; 6 Department of Medical Sciences, Psychiatry, Uppsala University, Uppsala, Sweden; 7 Department of Medicine Solna, Karolinska Institutet, Stockholm, Sweden; 8 Department of Cardiology, Karolinska University Hospital, Stockholm, Sweden; 9 Institute of Sport Science and Innovations, Lithuanian Sports University, Kaunas, Lithuania

**Keywords:** Psychosis, Biomarkers

## Abstract

Patients with schizophrenia have a threefold higher mortality from cardiovascular disease than people in the general population. Atherosclerosis is linked to immune activation, a process tentatively entwined with the underlying pathophysiological mechanisms of schizophrenia. The aim of the present study was to investigate an extensive array of cardiovascular biomarkers in individuals experiencing their first episode of psychosis (FEP), either drug-naïve or exposed to short-term antipsychotic treatment, alongside a group of healthy controls (HC). Using the OLINK Proximity Extension Assay, Cardiovascular II Panel, we analyzed plasma from 72 FEP patients, including 42 later diagnosed with schizophrenia and 54 HCs. Biomarker levels, that significantly differed between patients and controls, were correlated with symptom severity, cognitive performance and cardiovascular risk factors. Fifteen out of 92 cardiovascular biomarkers were higher in individuals with FEP compared to HC, and one biomarker was lower in FEP patients compared to HC. BMI, waist size, blood pressure, fp-glucose, HbA1c and serum lipid levels were similar between the groups. No correlations that held for multiple comparisons were seen between biomarker concentrations and symptom severity, cognitive performance or cardiovascular risk factors in FEP patients. Higher concentrations of several cardiovascular biomarkers were observed in individuals with FEP compared to in HC. This suggests that patients with FEP are at an increased risk of developing cardiovascular disease from the onset of psychosis, even before changes in traditional biomarkers are detectable. It underscores the need for innovative approaches to detect and monitor this risk early.

## Introduction

Schizophrenia is a disorder affecting ~0.5% of the population and their families worldwide^1^. The clinical diagnosis is defined by a wide range of symptoms, including hallucinations, delusions, disorganized speech, diminished emotional expression, and avolition^2^. Additionally, individuals with schizophrenia typically display cognitive dysfunctions which can manifest already at the first episode of psychosis, before initiation of antipsychotic treatment^3,4^.

Beyond the disabling psychiatric symptoms, individuals with schizophrenia face a life expectancy 15–20 years shorter than the general population^5–7^. While the suicide rate is around 4.5 times higher than in the general population^8^, the primary cause of death in schizophrenia is cardiovascular disease, with an alarming mortality rate nearly three times higher than in the general population^9^. Coronary heart disease, including acute myocardial infarction and cerebrovascular disease, are the two most frequent cardiovascular causes of death^9^. Lifestyle factors that contribute to this disparity include heavy smoking, obesity, unhealthy diet, and inadequate physical activity^10–13^.

Additionally, antipsychotic medications, while essential for managing symptoms, can lead to weight gain^14–16^, hyperlipidemia^17^ and diabetes mellitus type II^18,19^, i.e., factors associated with cardiovascular disease. However, intriguingly, large epidemiological studies suggest that medication with antipsychotic drugs in schizophrenia is associated with a reduced risk of all-cause and cardiovascular mortality^20,21^.

Schizophrenia and cardiovascular diseases are both recognized as multifactorial conditions with complex and overlapping etiopathophysiologies. For example, both epidemiological studies^22,23^, genetic investigations^24^, experimental cell studies^25^ and biomarker analysis in plasma and cerebrospinal fluid (CSF)^26,27^ have pointed toward a role of immune factors in the progression of schizophrenia. Immune-related mechanisms also play a prominent role in the pathophysiology of atherosclerosis^28–30^ and several studies have shown associations between cardiovascular risk factors and psychotic symptoms and cognitive impairment in patients with schizophrenia^31,32^.

Building on this connection, we hypothesized that shared inflammatory aberrations might contribute to both the psychotic symptoms and the increased CVD vulnerability observed in patients with schizophrenia.

To explore the nature of the association between schizophrenia and cardiovascular disease further, we here focused on cardiovascular biomarkers in plasma using the OLINK cardiovascular panel II proximity extension assay (PEA) technology. We examined a well-characterized cohort of FEP patients who were either drug-naïve or had received short-term antipsychotic medication, alongside a group of healthy controls (HC).

Our objectives were twofold; first to investigate whether cardiovascular biomarkers differed between FEP patients and HC and FEP patients that later developed schizophrenia or schizoaffective disorder and healthy controls. Second, we aimed to assess whether levels of cardiovascular biomarkers measured by OLINK correlated with clinical ratings, cognitive function and cardiovascular risk factors in patients with FEP.

## Methods

### Subject population

This study was part of the Karolinska Schizophrenia Project (KaSP), a multidisciplinary research consortium that investigates the pathophysiology of schizophrenia. The study was conducted according to the Declaration of Helsinki principles and was approved by the Stockholm Regional Ethics Committee (2010/879-31-1). All participants were included between March 2011 and March 2019, and a signed consent was obtained from all participants.

### Patients with first episode psychosis

Seventy-three consecutive patients with FEP seeking health care for psychotic symptoms for the first time were recruited from psychiatric emergency wards and in- and outpatient facilities of five psychiatric clinics in Stockholm. Exclusion criteria were treatment with antipsychotic medication for >30 days, severe somatic or neurological disease, current or previous substance abuse (except nicotine use) and coexisting neurodevelopmental comorbidities such as autism-spectrum disorders.

The patients were diagnosed according to DSM-IV based on a structured clinical interview (SCID-I) or a consensus diagnostic procedure based on medical records supervised by an experienced psychiatrist (LF, SC, or CMS). At the time of inclusion, included patients either met the diagnostic criteria for schizophrenia (*n* = 22), schizoaffective disorder (*n* = 1), schizophreniform disorder (*n* = 23), brief psychotic disorder (*n* = 3), delusional disorder (*n* = 3), psychosis not otherwise specified (*n* = 18), or severe depression with psychotic features (*n* = 1). Two patients did not want to participate in the SCID-I interview and therefore did not receive specific diagnoses.

Fifty-six of the patients had their diagnosis reassessed, either with SCID-I or a consensus diagnostic procedure based on medical records after a 1.5-year follow-up. These patients then met the criteria for the diagnoses of schizophrenia (*n* = 38), schizoaffective disorder (*n* = 4), brief psychotic disorder (*n* = 1), delusional disorder (*n* = 3), psychosis not otherwise specified (*n* = 4) or mood disorder NOS (*n* = 1). Five of the patients had no psychosis-spectrum diagnosis after 1.5 years. These patients were then divided into two groups; one group of FEP patients with a diagnosis of schizophrenia or schizoaffective disorder (SCZ) and one group of FEP patients without a diagnosis of schizophrenia or schizoaffective disorder (non-SCZ).

Patients and close relatives provided information on the duration of untreated psychosis (DUP).

The patients underwent clinical characterization using the positive and negative syndrome scale (PANSS), the global assessment of functioning (GAF), and clinical global impression (CGI) and all raters attended repeated training sessions.

The Measurement and Treatment Research to Improve Cognition in Schizophrenia (MATRICS) Consensus Cognitive Battery^33^ was used to evaluate cognitive function. This includes ten tests that measure seven cognitive domains: Speed of processing (Brief Assessment of Cognition in Schizophrenia: Symbol Coding, Category Fluency: Animal Naming, Trail Making Test: Part A); Attention/vigilance (continuous performance test-identical pairs); Working memory (Wechsler Memory Scale-3rd Edition: Spatial Span, Letter-Number Span); Verbal learning (Hopkins Verbal Learning Test-Revised); Visual learning (brief visuospatial memory test-revised); Reasoning and problem solving (Neuropsychological Assessment Battery: Mazes) and Social cognition (Mayer–Salovey–Caruso Emotional Intelligence Test: Managing Emotions).

All patients underwent clinical examination, including neurological examination, measurement of blood pressure, BMI and waist circumference, routine laboratory tests and MRI examination of the brain, evaluated by an experienced neuroradiologist at the MR Centre, Karolinska University Hospital, Solna. Nicotine use was allowed, and patients were asked about their use of nicotine and smoking habits. Substance use was ruled out through the alcohol use disorders identification test (AUDIT), drug use disorders identification tests (DUDIT), drug-screening of urine and review of medical records.

The treating clinicians decided the patients’ medication, and 39 out of 73 patients were treated with antipsychotics at the time of plasma sampling (information is missing for one patient). Patients on antipsychotic medication used olanzapine, aripiprazole, risperidone, quetiapine, haloperidol, or levomepromazine. For full details of the patients’ medication, see Supplementary Table S1.

Clinical ratings (PANSS, GAF, and CGI), cognitive testing, and blood sampling was performed within 10 days for 58 FEP patients and within a time span of 11–57 days for 15 FEP (median 7 [IQR:5-10] days, *n* = 73). P-glucose, HbA1c, and s-lipids were either analyzed at the clinical laboratory on the day of blood sampling or drawn from medical records within a month of the time of inclusion.

### Healthy control subjects

Fifty-five HC living in Stockholm were recruited by advertisement. Exclusion criteria were neurologic disease, severe somatic disease, current or previous use of illegal substances and first-degree relatives with psychotic or bipolar disorder. This was excluded by clinical interview, clinical examination, review of medical history and the Mini International Neuropsychiatric Interview (M.I.N.I.). The HC underwent the same cognitive testing procedure as the patients.

All HCs underwent clinical examination, including neurological examination, measurement of blood pressure, BMI and waist circumference, routine laboratory tests and MRI examination of the brain, evaluated by an experienced neuroradiologist at the MR Centre, Karolinska University Hospital, Solna. One individual exhibited signs of demyelinating disease on MRI and oligoclonal bands in CSF, but as the clinical neurological exam was normal and there was no history of relevant neurological symptoms, the subject did not fulfill criteria for multiple sclerosis or any other clinically isolated syndrome. Therefore, this subject was not excluded from the analysis.

Smoking and nicotine use was permitted, and HCs were asked about their nicotine and smoking habits. Plasma sampling, clinical examination and cognitive testing were performed within a median of 24 (IQR:10–49) days.

### Plasma collection

Collection of peripheral blood was performed between 7:45 and 10 am for 53 FEP patients and 47 HC, and between 10 am and 1:15 pm for 16 FEP and six HC. For two HC and four FEP patients, information on the time point of blood sampling is missing. The subjects were instructed to avoid physical activity during the preceding 8 h. Peripheral venous blood was collected in 10 mL tubes containing EDTA (BD Vacutainer®; BD Hemograd, K2 EDTA) using standard venipuncture techniques. Samples were centrifuged at 2900 rpm for 15 min within 1 h of collection and stored at −80 °C until analysis.

### Proximity extension assay

The samples were analyzed at the Olink Clinical Biomarkers Facility, Science for Life Laboratory, Uppsala University, Sweden, using the Olink Cardiovascular II panel which is a panel consisting of 92 preselected cardiovascular disease-related protein biomarkers (See Supplementary Table S2). Olink uses proximity extension assay (PEA) technology, where pairs of antibodies with unique oligonucleotide tags simultaneously bind to their target protein in the sample. When the matched DNA are brought into proximity, they start to hybridize, resulting in a reporter sequence that can be quantified through real-time PCR, where the number of qPCR samples is related to the concentration of the protein. The PCR results are then normalized using internal and external controls, and relative concentrations for all biomarkers are reported in a log2 unit called Normalized Protein eXpression (NPX). The limit of detection (LOD) was reported by OLINK for each biomarker. More information is available from www.olink.com.

### Statistical analysis

The normality of data were determined using the Shapiro–Wilk test. Demographic data, clinical scores and cardiovascular risk factors were presented as median (IQR) and compared between FEP patients and HC; SCZ and HC and non-SCZ and HC using the Mann–Whitney *U*-test for continuous variables and the chi-square test for categorical variables.

Concentrations of one protein, BNP, was below the protein-specific limit of detection (LOD) in ~90% of the samples and was therefore removed from further analyzes. Samples with NPX values below the protein-specific LOD value were treated as missing values.

Outlier detection was done performing OlinkAnalyze package in R where the standard deviations from the mean interquartile range and mean sample median for every sample is checked. Additionally, a principal component analysis (PCA) was performed to see whether any samples deviated from the overall distribution pattern. Outliers were defined as samples with an average NPX below 1.5 first quartile or above 1.5 third quartile, and no outliers were found.

To identify differentially expressed proteins between groups, the limma package^34^ was used to apply multiple linear models. Limma is a powerful package^35^ for identifying differentially expressed proteins, which employs an empirical Bayes method, that provides an advantage in moderating the standard errors of residual variances. Differential expression was calculated for FEP patients compared to HC and SCZ, and non-SCZ separately compared to HC. PCA was performed to identify samples deviating from the overall distribution pattern and potential confounders. PCA analyses were done with “OlinkAnalyze” package in R, using the built-in “olink_pca_plot” function (www.olink.com) and samples were analyzed by their plate, age, gender, and nicotine usage to see if any of these variables were potential confounders. Since levels of biomarkers differed between samples on plate 1 and plate 2, analyses were adjusted for plate number. To control for multiple testing, we applied the Benjamini–Hochberg correction^36^ with a false discovery rate (FDR) of 0.05. This method adjusted for 91 statistical tests in the group comparisons of FEP and HC, SCZ, and HC and non-s. Additionally, we included age, gender, and nicotine use in the final analysis, to account for potential confounding factors.

In FEP patients and FEP patients later diagnosed with schizophrenia or schizoaffective disorder separately, biomarker levels that showed significant differences compared to in HCs were correlated with disease severity scores (PANSS, CGI, and GAF), cognitive ratings, BMI, waist size, blood pressure, p-glucose, HbA1c, and lipid levels using Spearman correlation analyses. The analyses were adjusted for multiple comparisons using the Benjamini–Hochberg correction method.

To assess other potential confounders, demographic data were compared between groups and expression of proteins was compared between FEP patients using antipsychotic medication and drug-naïve patients.

All reported *p*-levels are two-sided, and statistical significance was considered when *p* ≤ 0.05. All analyses were performed using RStudio (version 1.3.1093) with R (version 4.1.0), Prism (GraphPad Software, version 9.0) or SPSS (IBM Statistics, version 28.0).

## Results

### Demographics

Two samples (one FEP patient and one HC) did not pass OLINK’s quality control and were subsequently excluded from further analysis. Table 1 provides a summary of demographics and clinical ratings for the remaining 72 FEP patients (42 males and 30 females) and 54 healthy controls (26 males and 28 females). Additionally, Supplementary Table S3 presents these details for the 56 FEP patients who were stratified by patient group (SCZ or non-SCZ) at the 1.5-year follow-up. No significant differences in age, BMI, smoking or gender were found between groups. Nicotine use was borderline significantly different between FEP patients and HC (*p* = 0.08) and significantly different between HC and non-SCZ patients (*p* = 0.01). Median DUP of the patients was 4.5 (IQR: 1–12) months, which was significantly longer in SCZ patients as compared to non-SCZ (6 [IQR:2–18] months vs. 3 [IQR 1–5**]** months, *p* = 0.03). Fifty-five percent of the FEP patients were medicated with antipsychotics. In the SCZ patient group, 62% were medicated with antipsychotics versus 14% in the non-SCZ group.

**Table 1:** Demographics and characteristics of patients with first episode psychosis and healthy controls.

Characteristic	Median [IQR] (nr)		*p* value
	HC^54^	FEP^72^	
Gender, male/female	26/28^54^	42/30^72^	0.35^a^
Age, years	26 [23–30]^54^	28.0 [23–33]^72^	0.33^b^
BMI, kg/m^−2^	23.0 [21.0–26.0]^51^	22.05 [21.0–25.0]^70^	0.15^b^
Nicotine use, %	13^53^	26^69^	0.08^a^
Smoking, %	4^53^	10^71^	0.19^a^
DUP, months	-	4.5 [1.0–12.0]^60^	-
Medication, nr			
Antipsychotics	0^54^	39^71^	-
Antidepressants	0^54^	7^71^	-
Benzodiazepines/Benzo-diazepine-like drugs	0^54^	28^71^	-
Phenothiazine derivates	0^54^	14^71^	-
Mood-stabilizers	0^54^	2^71^	-
PANSS			
Positive	-	18 [13–23]^71^	-
Negative	-	17 [12–22]^71^	-
General	-	35 [28–44]^71^	-
Total	-	72 [59–85]^71^	-
Level of functioning	-		
CGI	-	5 [4–5]^71^	-
GAF symptoms	-	32 [27–40]^70^	-
GAF function	-	40 [34–50]^70^	-

*BMI* body mass index, *DUP* duration of untreated psychosis, *PANSS* positive and negative syndrome scale, *CGI* clinical global impression, *GAF* global assessment of functioning.

^a^Chi-square test.

^b^Mann–Whitney *U*-test.

### OLINK cardiovascular protein profiling in the plasma of patients with first episode psychosis vs. in healthy controls

Out of 92 biomarkers, 80 were detectable in over 90% of the plasma samples, and seven were detected in 70–89% of the samples. Average expression, log_2_ fold change, percent samples over LOD and *p* value adjusted for plate and multiple comparisons of all 92 biomarkers in the OLINK cardiovascular panel II in FEP patients and HC are shown in Supplementary Table S4.

**Fig. 1:**
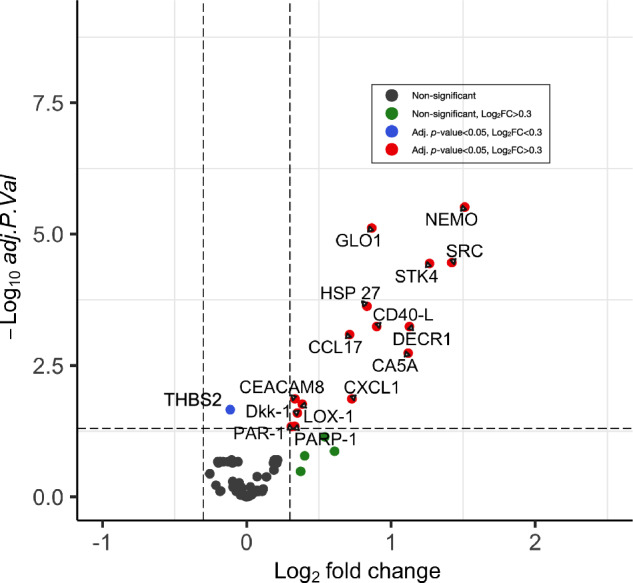
Differences in plasma cardiovascular proteins in patients with first episode psychosis vs. healthy controls. Ninety-one biomarkers are presented in a volcano plot with log_2_ fold change between FEP patients and HC. Log_2_ fold changes between groups and *p* values are calculated with the limma package in R and adjusted for plate, age, gender, and nicotine use. Adjustment for multiple comparisons was performed using the Benjamini–Hochberg correction method and was corrected for 91 tests. Significance level chosen for adjusted *p* value is <0.05.

**Fig. 2:**
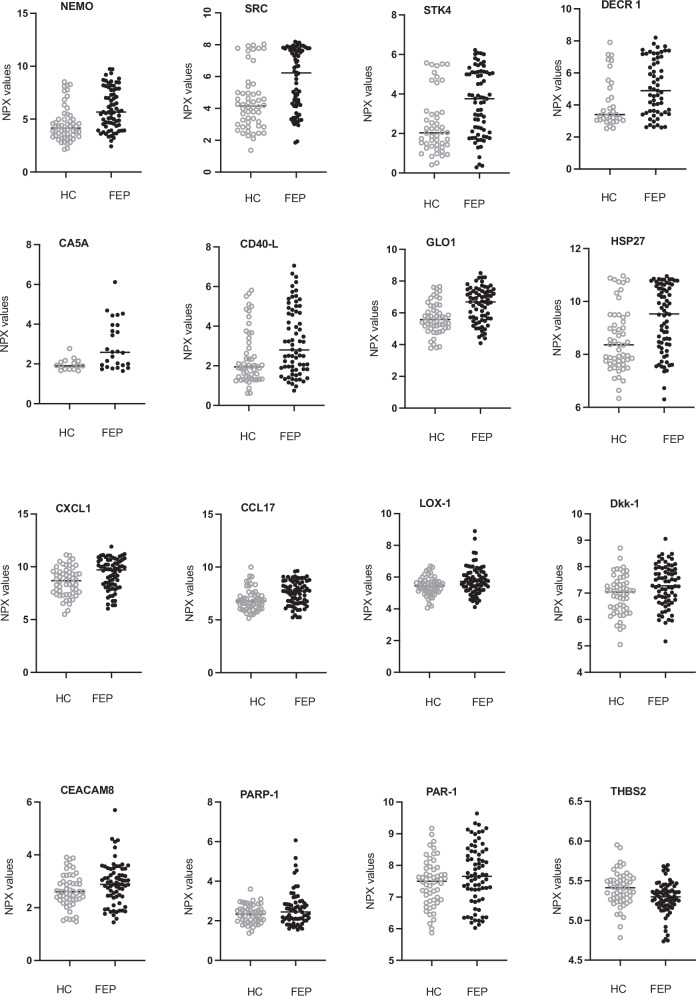
OLINK cardiovascular protein profiling in plasma of patients with first episode psychosis vs. healthy controls. Scatter dot plots display 16 biomarkers that show significant differences between FEP patients and HC. The horizontal line represents the median value. HC healthy controls, FEP first episode psychosis patients.

Upon conducting a differential expression analysis between FEP patients and HC, while accounting for plate number, gender, age, nicotine use and multiple comparisons, we identified 15 biomarkers (NEMO, SRC, STK4, DECR1, CA5A, CD40-L, GLO1, HSP-27, CXCL1, CCL17, LOX-1, Dkk-1, CEACAM8, PARP-1, and PAR-1) that exhibited significantly higher concentrations in FEP patients compared to HC. NEMO displayed the highest log_2_ fold change (log_2_ FC 1.51, adj. *p* value 0.0000031) followed by SRC (log_2_ FC 1.42, adj. *p* value 0.000035), STK4 (log_2_ FC 1.27, adj. *p* value 0.000036 and DECR1 (log_2_ FC 1.13, adj. *p* value 0,00057). The protein THBS2 was lower in FEP patients compared to HC (log_2_ FC −0.11, adj. *p* value 0.015). Significantly different protein concentrations between FEP patients and HC are shown in a volcano plot in Fig. 1 and in scatter dot plots in Fig. 2. Protein expression of these 16 biomarkers did not differ significantly between drug-naïve FEP patients and FEP patients on antipsychotic treatment (see Supplementary Table S5). Log_2_ fold change and *p* value adjusted for plate, age, gender, nicotine usage, and multiple comparisons of all 91 biomarkers are shown in Supplementary Table S6. When adding freezing time as an additional confounder, the log fold change between FEP and HC were still significant for the five biomarkers with the largest log fold change (NEMO, GLO1, CA5A, STK4, and SRC).

### OLINK cardiovascular protein profiling in plasma of patients with first episode psychosis later diagnosed with schizophrenia or schizoaffective disorder vs. healthy controls

Differential expression analysis between SCZ patients (*n* = 42) and HC (*n* = 54) showed that 16 proteins (SRC, NEMO, STK4, DECR1, CD40-L, HSP-27, CXCL1, GLO1, CA5A, CCL17, PDGF subunit B, LOX-1, Dkk-1, PAR-1, CEACAM8, and CD84) were significantly higher and one protein, THBS2, was significantly lower (log_2_ FC −0.15 adj. *p* value 0.0060) in SCZ patients than in HC, after correction for age, gender, nicotine use, plate number and multiple comparisons, which is shown in Supplementary Table S7. SRC displayed the highest log_2_ fold change (log_2_ FC 1.99, adj. *p* value 0.000000078) followed by NEMO (log_2_ FC 1.96, adj. *p* value 0.000000044) and STK4 (log_2_ FC 1.69 adj. *p* value 0.00000059). Log_2_ fold change and *p* value adjusted for plate, age, gender, nicotine usage, and multiple comparisons of all 91 biomarkers are shown in Supplementary Table S8.

### OLINK cardiovascular protein profiling in plasma of patients with first episode psychosis later not diagnosed with schizophrenia vs. healthy controls

No biomarkers were significantly different in plasma between non-SCZ patients (*n* = 14) and HC (*n* = 54) after adjusting for age, gender, nicotine use, plate and multiple comparisons.

### Correlations of OLINK cardiovascular biomarkers and clinical rating scores and cognitive measures in patients with first episode psychosis

**Fig. 3:**
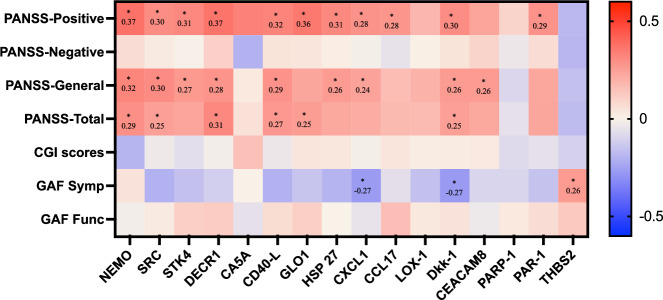
Correlations of significantly different biomarkers in plasma (NPX values) and clinical rating scores (PANSS, GAF, and CGI) in patients with first episode psychosis. Correlation analysis was performed using Spearman correlation (r_s_) and numbers in the heat map boxes indicate r_s_ values. The color represents the value of the Spearman’s rank coefficient, and red is a positive correlation and blue a negative correlation. The asterisk indicates a significant correlation (*p* < 0.05). None of the correlations remained significant after correction for multiple analysis with Benjamini–Hochberg’s method. PANSS positive and negative syndrome scale, CGI clinical global impression, GAF global assessment of functioning.

**Fig. 4:**
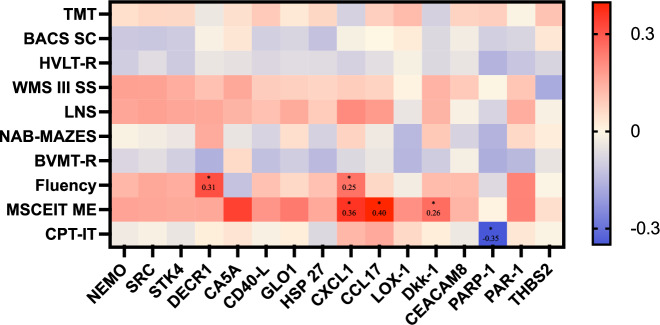
Correlations of significantly different biomarkers in plasma (NPX) and cognitive measures (MATRICS) in patients with first episode psychosis. Correlation analysis was performed using Spearman correlation (r_s_) and numbers in the heat map boxes indicate r_s_ values. The color represents the value of the Spearman’s rank coefficient, and red is a positive correlation and blue a negative correlation. The asterisk indicates a significant correlation (*p* < 0.05). None of the correlations remained significant after correction for multiple analysis with Benjamini–Hochberg’s method. TMT trail making test, Part A, BACS SC brief assessment of cognition in schizophrenia, symbol coding, HVLT-R Hopkins verbal learning test-revised, WMS III SS Wechsler memory scale–3rd edition: spatial span, LNS letter-number span, NAB-MAZES neuropsychological assessment battery: Mazes, BVMT-R brief visuospatial memory test revised, Fluency category fluency: animal naming, MSCEIT ME Mayer–Salovey–Caruso emotional intelligence test: managing emotions, and CPT continuous performance test-identical pairs.

In FEP patients, we examined correlations between the 16 biomarkers (NEMO, SRC, STK4, DECR1, CA5A, CD40-L, GLO1, HSP-27, CXCL1, CCL17, LOX-1, Dkk-1, CEACAM8, PARP-1, PAR-1, and THBS2) that showed significant differences compared to HCs and clinical ratings (PANSS, CGI, and GAF), as illustrated in Fig. 3, and cognitive measures (MATRICS Consensus Cognitive Battery), as presented in Fig. 4.

Higher biomarkers correlated with higher PANSS positive scores for 12 biomarkers, with higher PANSS negative scores for nine biomarkers and with higher PANSS total scores for six biomarkers. Higher CXCL correlated with lower GAF symptom score. The strongest correlations with clinical ratings were seen between NEMO and PANSS pos (r_s_ = 0.372, *p* = 0.001), DECR1 and PANSS pos (r_s_ = 0.369, *p* = 0.004), GLO1 and PANSS pos (r_s_ = 0.356, *p* = 0.002) and CD40-L and PANSS pos (r_s_ = 0.324, *p* = 0.006). THBS2 was significantly lower in FEP patients compared to HC, and lower levels correlated with lower GAF function scores. However, none of these correlations remained significant after adjusting for multiple comparisons.

Regarding cognitive functioning in FEP patients; higher biomarkers correlated with higher cognitive scores for DECR1 and Fluency (r_s_ = 0.312, *p* = 0.02), CXCL1 and Fluency (r_s_ = 0.252, *p* = 0.038), CXCL1 and MSCEIT ME (r_s_ = 0.359, *p* = 0.003) CCL17 and MSCEIT ME (rs = 0.397, *p* = 0.001) and Dkk-1 and MSCET ME (r_s_ = 0.261, *p* = 0.36). Higher PARP-1 levels were correlated with lower cognitive scores on the CPT-IT (r_s_ = −0.350, *p* = 0.008). None of the correlations remained significant after corrections for multiple analysis.

### Cardiovascular parameters in patients with first episode psychosis and healthy controls

Cardiovascular parameters in FEP patients and HC are presented in Table 2. Diastolic blood pressure was slightly higher in patients with FEP as compared to in HCs (80 [IQR: 70–80] vs 70 [IQR:70-80] *p* = 0.01). The other parameters were similar between the groups.

**Table 2:** Cardiovascular risk factors in patients with first episode psychosis and healthy controls.

Characteristic	Median [IQR] (nr)		*p* value HC vs FEP
	Healthy controls (*n* = 54)	FEP (*n* = 72)	
Blood pressure systolic, mmHg	115 [110–120]^53^	120 [110–130]^68^	0.13^a^
Blood pressure diastolic, mmHg	70 [70–80]^53^	80 [70–80]^67^	0.01^a^
Waist circumference, cm	83 [72–89]^43^	83 [72–89]^60^	0.74^a^
Fasting p-glucose, mmol/L	5.3 [5.1–5.4]^19^	5.0 [4.9-5.6]^31^	0.09^a^
B-HbA1c, mmol/mol	33.0 [30.0–36.0]^19^	31.0 [29.0-34.0]^15^	0.24^a^
P-cholesterol, mmol/L	4.10 [3.60–4.70]^19^	4.50 [4.00–5.10]^29^	0.12^a^
P-LDL, mmol/L	2.40 [2.00–2.60]^19^	2.55 [2.20–3.08]^28^	0.17^a^
P-HDL, mmol/L	1.50 [1.20–1.70]^19^	1.30 [1.15–1.65]^29^	0.33^a^
P-LDL/HDL ratio	1.40 [1.25–2.08]^19^	1.90 [1.41–2.45] 28)	0.08^a^
P-triglycerides, mmol/L	0.60 [0.59–0.89]^19^	0.90 [0.66–1.13]^26^	0.10^a^

^a^Mann–Whitney *U*-test.

*HDL* high-density lipoprotein, *LDL* low-density lipoprotein.

### Associations of OLINK cardiovascular biomarkers and cardiovascular risk factors in patients with first episode psychosis

In individuals with FEP, we analyzed correlations of the 16 biomarkers (NEMO, SRC, STK4, DECR1, CA5A, CD40-L, GLO1, HSP-27, CXCL1, CCL17, LOX-1, Dkk-1, CEACAM8, PARP-1, PAR-1, and THBS2) that exhibited significant differences when compared to HC and known cardiovascular risk factors, including BMI, waist circumference, systolic and diastolic blood pressure, fasting plasma glucose, HbA1c, p-cholesterol, p-LDL, p-HDL, P-LDL/HDL, and p-triglycerides. Correlations were observed between higher systolic blood pressure and increased levels of NEMO, SRC, STK4, CD40-L, GLO1, HSP-27, Dkk-1, PARP-1, and PAR-1, as well as between higher NEMO and a higher LDL/HDL ratio, and between higher HDL and lower NEMO, SRC, and STK4. However, these associations did not remain significant after correction for multiple comparisons. The findings are presented in Supplementary Table S9.

## Discussion

In this cross-sectional study, we investigated the proteomic plasma profile of FEP patients and HC using the OLINK proximity extension assay, specifically the Cardiovascular II Panel. Our key finding is that the proteomic plasma profile of cardiovascular biomarkers significantly differs between FEP patients and HCs. Notably, we identified 16 biomarkers that exhibited significant differences between FEP patients and HCs. No differences were seen between patients who were drug-naïve and patients with short-term antipsychotic treatment. Interestingly, differences compared to HCs were more pronounced in FEP patients who later received a diagnosis of schizophrenia or schizoaffective disorder as compared to those who did not fulfill the criteria for these disorders.

Associations between the significantly different biomarkers and clinical ratings and cognitive measures were not statistically significant after correcting for multiple comparisons with Benjamini–Hochberg’s method. A recent meta-analysis found that metabolic syndrome, diabetes and hypertension are associated with cognitive impairment in schizophrenia. Notably, our study focused on younger FEP patients with a shorter illness duration (mean age 28 years, DUP 4.5 months) compared to those in the meta-analysis (mean age 43 years, illness duration 14 years). This suggests that cognitive association with cardiovascular risk factors may develop over time, underscoring the importance of longitudinal studies to clarify when these risks begin to affect cognition in psychosis.

While the diastolic blood pressure was slightly higher among FEP subjects, traditional cardiovascular risk factors were overall remarkably similar between FEP patients and HCs. However, this finding is in contrast with other studies that have reported increased rates of metabolic syndrome, higher BMI, elevated glucose, and altered cholesterol levels in FEP patients^37–40^. Several factors may contribute to this discrepancy. Firstly, our study had a relatively small number of participants with available information on cardiovascular risk factors. Additionally, our cohort consisted of young individuals newly diagnosed with psychosis, and only a minority of FEP patients (10%) and HC (4%) reported smoking tobacco. Furthermore, patients with comorbid substance use disorder were excluded, and it is well established that cardiovascular disease and all-cause mortality are higher in this group^41,42^.

Despite the similarity between the groups in traditional cardiovascular risk factors, differences in cardiovascular proteomics profiling were evident between FEP patients and HC. These findings suggest that early cardiovascular changes in FEP patients may not be detectable using conventional examinations.

The most pronounced difference observed between FEP patients and HC was in NEMO (Nuclear factor-κB Essential Modulator), which is broadly expressed in both the brain and peripheral tissues, such as hepatocytes and cardiomyocytes. NEMO is crucial for the activation of NF-κB, a protein complex known to regulate the transcription of various immune signaling molecules such as cytokines, chemokines, and immune receptors^43,44^. There are several studies showing dysregulation of NF-κB in schizophrenia disorder^45–47^. Postmortem brain analyses have revealed downregulation of the entire NF-κB system, especially in the temporal cortex, in individuals with schizophrenia^48^. Later studies support a role of the NF-κB pathway in the manifestation of schizophrenia symptoms, based on overactivity of this pathway in the dorsolateral prefrontal cortex^49,50^, an area implicated in cognitive and executive functions, in patients with schizophrenia. Another study showed a statistical trend, even though not significant, toward increased expression of NF-κB in monocytes of patients with schizophrenia^51^. Altogether, while NEMO-specific studies in schizophrenia are lacking, the NF-κB pathway remains a promising area of investigation in understanding the molecular underpinnings of schizophrenia.

The NF-κB pathway has also been implicated in various CVDs, including atherosclerosis, myocardial infarction, heart failure and ischemic stroke^43^.

Experimental studies have shown that endothelium-restricted inhibition of NF-κB activation, including ablation of NEMO, resulted in significantly reduced atherosclerotic plaque formation in mice^52^ and that ablation of NEMO in smooth muscle cells (SMC) inhibited high-fat diet-induced atherosclerosis in mice^53^. Further, inhibiting NF-κB using pharmacological inhibitors suppresses myocardial injury following ischemia^54–56^ and leads to improved cardiac function and lower mortality after myocardial infarction in mice^57,58^. However, according to experimental studies it appears that NF-κB has dual effects on myocardial injury following ischemia^43,59^. Thus, inhibition of the NF-κB pathway in macrophages leads to more severe atherosclerosis in mice^60^, and further, NF-κB activation is protective against myocardial infarction^61^. Altogether, the functional significance of the NEMO-controlled activation of the NF-κB pathway with regard to CVD appears intricate, and the precise mechanisms by which it bidirectionally influences atherosclerosis and myocardial infarction protection are yet to be fully understood.

In all, our study indicates that the NF-κB pathway is a common pathophysiological player in both CVD and schizophrenia, potentially explaining the increased cardiovascular co-morbidity observed in patients with psychotic disorders. Further research into the role of NF-κB signalling in schizophrenia could provide valuable insights into therapeutic targets for reducing early mortality in this population.

Another protein that was found to be profoundly changed between cohorts was Src, a non-receptor protein–tyrosine kinase, which is expressed in various cell types, with the highest levels in the brain, osteoclasts, and platelets^62,63^. Notably, Src signaling plays a crucial role in the crosstalk between tumor and inflammatory cells^64^. A prior investigation demonstrated diminished Src signaling in postmortem tissue from individuals with schizophrenia, alongside decreased NMDAR signaling^65^ despite an elevated overall expression of NMDA receptors, suggesting that NMDAR dysfunction in schizophrenia may involve Src-induced hypofunction^65^. In another study, the effects of atypical antipsychotics and a Src inhibitor on cortical injury in rats were examined, with both antipsychotics and the Src inhibitor showing protective effects^66^. Furthermore, a separate study demonstrated the antitumor effects of aripiprazole, a commonly used atypical antipsychotic agent, and observed reduced levels and activity of Src following aripiprazole treatment^67^. Certainly, Src activation plays a pivotal role in various cancers, reducing survival and increasing risk of metastasis and consequently, Src inhibition is a promising avenue for the development of anticancer medications^67–69^. The documentation of the influence of Src on the development of CVD is relatively sparse, although Src activity has been associated with cardiac hypertrophy^70^ and hypertension-related mechanisms^71,72^. Altogether, the high levels of Src in FEP patients, may be another common denominator between schizophrenia and co-morbidity with respect to both CVD and cancer.

STK4, another aberrant cardiovascular biomarker, also known as the mammalian Sterile 20-like kinase 1 (MST 1), is a serine-threonine kinase that serves as a core component of the Hippo pathway, regulating organ size through the modulation of cell proliferation and apoptosis^73^. While studies on STK4 in schizophrenia are limited, research indicates its involvement in cardiovascular diseases such as aortic dissection, aortic aneurysm, atherosclerosis and myocardial ischemic injury^67^, as well as neurological and cerebrovascular diseases.^74^. Clearly, a role of this protein in the pathophysiology of schizophrenia remains to be elucidated.

The present study’s strengths lie in its inclusion of a relatively large cohort of well-characterized FEP patients, of which almost half were fully antipsychotic-naïve, with comprehensive data on clinical ratings, cognitive assessments, cardiovascular risk factors, medication usage and follow-up diagnoses. One limitation of this study is incomplete information on cardiovascular status for some participants. Additionally, the exclusion of patients with drug abuse might limit the generalizability of the findings, given that cannabis use is prevalent in around a third of individuals experiencing FEP^75^. However, this exclusion was necessary to mitigate the potential influence of substance abuse on biomarkers, aligning with the study’s focus on elucidating underlying pathophysiological mechanisms. A further limitation is the sensitivity to preanalytical sampling in some biomarkers, esp. SRC, CD40-L and HSP-27^76^. However, FEP patients and HC were collected in parallel, mixed over time, and all procedures such as sample collection, storage sites, freezing and thawing procedures and centrifugation, as well as the equipment utilized, were identical for both the control group and patients^77^.

In summary, our findings indicate significant differences in the proteomic plasma profiles of cardiovascular biomarkers between individuals experiencing FEP and HC, even when cardiovascular risk factors are similar between the groups. This underscores the necessity for novel approaches to detect and monitor cardiovascular risk in FEP patients, highlighting the potential for improved strategies in clinical management and risk assessment within this population.

## Supplementary information

Supplementory

## Data Availability

The data supporting the findings of this study are not publicly available due to ethical and legal restrictions related to participant confidentiality. Access to the data may be granted upon reasonable request to the corresponding author after approval by the Stockholm Regional Ethics Committee. The data are stored in a controlled-access repository at Karolinska Institutet.

## Code availability

The code used in this study can be obtained from the corresponding author upon request.
