# Supplementary material for: Cardiovascular protein profiling in patients with first-episode psychosis
Source: Schizophrenia (Heidelb). 2025 Jun 14;11(1):88. doi: 10.1038/s41537-025-00633-x (PMC12167380; doi:10.1038/s41537-025-00633-x)
Supplement: Supplementary file 1 — Supplementory [file 41537_2025_633_MOESM1_ESM.pdf]

Supplementary

S.1.

Medication of patients with first episode psychosis.

Patient nr	Antipsychotics (mg/day)	Antidepressants (mg/day)	Mood-stabilizers (mg/day)	Phentiazinederivates (mg/day)	Benzodiazepines/ Benzodiazepine-like drugs (mg/day)
1	-	-	-	-	-
2	-	-	-	-	Zopiclone (7,5)
3	-	-	-	-	-
4	Olanzapine (10), Quetiapine (600)	-	-	Alimemazine (60), Propiomazine (25)	Oxazepam (10)
5	Olanzapine (10)	Mirtazapine (30)	-	-	Diazepam (10), Zopiclone (7,5)
6	Olanzapine (10)	-	-	-	-
7	Risperidone (2)	-	-	-	Zopiclone (7,5)
8	Olanzapine (30)	-	-	-	-
9	Olanzapine (10)	-	-	-	-
10	-	-	-	Propiomazine (25)	Zolpidem (10)
11	Quetiapine (50)	Venlafaxine (300)	Lamotrigine (200)	-	Zopiclone (7,5)
12	-	-	-	-	Nitrazepam (5)
13	Olanzapine (10), Quetiapine (450)	-	-	Propiomazine (25)	-
14	-	-	-	-	-
15	Olanzapine (20)	-	-	Alimemazine (20)	-
16	-	Citalopram (20)	-	-	-
17	Risperidone (4)	-	-	-	-
18	Olanzapine (15)	-	-	-	-
19	Olanzapine (5)	-	-	-	-
20	-	-	-	-	Oxazepam (10)
21	-	-	-	-	Oxazepam (20)
22	Risperidone (4)	-	-	Promethazine (50), Propiomazine (50)	Zopiclone (7,5)
23	Olanzapine (5)	-	-	-	-
24	-	-	-	-	Oxazepam (5)
25	-	-	-	Propiomazine (25)	Oxazepam (15), Zopiclone (7,5)

26	Haloperidol (6), Olanzapine (5)	-	-	-	-
27	-	-	-	Propiomazine (25)	Oxazepam (15), Zopiclone (7,5)
28	-	-	-	-	Zopiclone (7,5)
29	-	-	-	Propiomazine (50)	Zopiclone (7,5)
30	Aripiprazole (10)	-	-	-	-
31	Olanzapine (15)	-	-	-	Zopiclone (7,5)
32	-	-	-	-	-
33	-	-	-	-	-
34	-	-	-	-	-
35	Olanzapine (15)	Citalopram (20)	-	-	Zopiclone (5)
36	Olanzapine (10)	-	-	-	Zolpidem (10)
37	Aripiprazole (10)	-	-	-	-
38	-	-	-	-	-
39	Aripiprazole (10)	-	-	-	Oxazepam (10)
40	Olanzapine (10)	-	-	Propiomazine (25)	Oxazepam (10), Nitrazepam (5)
41	-	-	-	-	-
42	Olanzapine (10)	-	-	Propiomazine (25)	-
43	-	-	-	-	-
44	Haloperidol (1), Olanzapine (15)	-	-	-	-
45	Risperidone (4)	Mirtazapine (30)	-	-	Oxazepam (10)
46	-	-	-	-	-
47	-	Paroxetine (20)	-	-	Oxazepam (10), Zopiclone (7,5)
48	-	-	-	-	-
49	-	-	-	-	-
50	Risperidone (4)	-	-	-	Oxazepam (10), Zopiclone (7,5)
51	-	-	-	-	-
52	Aripiprazole (10)	-	-	-	-
53	-	-	-	-	-
54	Olanzapine (20)	-	-	-	-
55	-	-	-	-	-
56	-	-	-	-	-
57	Olanzapine (10)	-	-	-	-

58	Olanzapine (10)	Sertraline (100)	-	-	-
59	Aripiprazole (5), Levo-meprazine (100), Olanzapine (15)	-	Lithionit (168)	-	-
60	-	-	-	-	-
61	Aripiprazole (10), Olanzap-ine (10)	-	-	-	Zopiclone (7,5)
62	Risperidone (1)	-	-	-	-
63	-	-	-	-	-
64	Aripiprazole (20)	-	-	-	Oxazepam (10), Zopiclone (7,5)
65	Aripiprazole (10)	-	-	Propiomazine (50)	-
66	Aripiprazole (15)	-	-	Alimemazine (40)	-
67	Olanzapine (20)	-	-	-	-
68	Aripiprazole (15)	-	-	Promethazine (75)	Zopiclone (5)
69	-	-	-	-	Nitrazepam (5)
70	-	-	-	Propiomazine (50)	Zopiclone (7,5)
71	Risperidone (10)	-	-	-	-
72	Unknown	Unknown	Unknown	Unknown	Unknown

S.2.

Biomarkers of OLINK Cardiovascular II panel

**Protein**	**Abbreviation**
2,4-dienoyl-CoA reductase, mitochondrial (DECR1)	DECR 1
A disintegrin and metalloproteinase with thrombospondin motifs	ADAM-TS13
ADM (ADM)	ADM
Agouti-related protein	AGRP
Alpha-L-iduronidase	IDUA
Angiopoietin-1	ANG-1
Angiopoietin-1 receptor	TIE2
Angiotensin-converting enzyme 2	ACE2
Bone morphogenetic protein 6	BMP-6
Brother of CDO	BOC
C-C motif chemokine 17	CCL17
C-C motif chemokine 3	CCL3
C-X-C motif chemokine 1	CXCL1
Carbonic anhydrase 5A, mitochondrial	CA5A
Carcinoembryonic antigenrelated cell adhesion molecule 8	CEACAM8
Cathepsin L1	CTSL1
CD40 ligand	CD40-L
Chymotrypsin C	CTRC
Decorin (DCN)	DCN
Dickkopf-related protein 1 (Dkk-1)	Dkk-1
Fatty acid-binding protein, intestinal	FABP2
Fibroblast growth factor 21	FGF-21
Fibroblast growth factor 23	FGF-23
Follistatin	FS
Galectin-9	Gal-9
Gastric intrinsic factor	GIF
Gastrotropin	GT
Growth hormone	GH
Growth/differentiation factor 2	GDF-2
Heat shock 27 kDa protein	HSP 27
Heme oxygenase 1	HO-1
Hydroxyacid oxidase 1	HAOX1
Interleukin-1 receptor antagonist protein	IL-1ra
Interleukin-1 receptor-like 2	IL1RL2
Interleukin-17D	IL-17D
Interleukin-18	IL-18
Interleukin-27	IL-27

Interleukin-4 receptor subunit alpha	IL-4RA
Interleukin-6	IL6
Kidney Injury Molecule	KIM1
Lactoylglutathione lyase	GLO1
Lectin-like oxidized LDL receptor 1	LOX-1
Leptin	LEP
Lipoprotein lipase	LPL
Low affinity immunoglobulin gamma Fc region receptor II-b	IgG Fc receptor II-b
Lymphotactin	XCL1
Macrophage receptor MARCO	MARCO
Matrix metalloproteinase-12	MMP-12
Matrix metalloproteinase-7	MMP-7
Melusin	ITGB1BP2
Natriuretic peptides B	BNP
NF-kappa-B essential modulator	NEMO
Osteoclast-associated immunoglobulin-like receptor	hOSCAR
P-selectin glycoprotein ligand 1	PSGL-1
P16860 Serine protease 27	PRSS27
Pappalysin-1	PAPPA
Pentraxin-related protein	PTX3
Placenta growth factor	PGF
Platelet-derived growth factor subunit B	PDGF subunit B
Poly [ADP-ribose] polymerase 1	PARP-1
Polymeric immunoglobulin receptor	PIgR
Pro-interleukin-16	IL16
Programmed cell death 1 ligand 2	PD-L2
Proheparin-binding EGF-like growth factor	HB-EGF
Prolargin	PRELP
Prostasin	PRSS8
Protein AMBP	AMBP
Protein-glutamine gamma-glutamyltransferase 2	TGM2
Proteinase-activated receptor 1	PAR-1
Proto-oncogene tyrosine-protein kinase Src	SRC
Receptor for advanced glycosylation end products	RAGE
Renin	REN
Serine/threonine-protein kinase 4	STK4
Serpin A12	SERPINA12
SLAM family member 5	CD84
SLAM family member 7	SLAMF7
Sortilin	SORT1
Spondin-2	SPON2
Stem cell factor	SCF
Superoxide dismutase mitochondrial	SOD2
T-cell surface glycoprotein CD4	CD4

Thrombomodulin	TM
Thrombopoietin	THPO
Thrombospondin-2	THBS2
Tissue factor	TF
TNF-related apoptosis-inducing ligand receptor 2	TRAIL-R2
Tumor necrosis factor receptor superfamily member 10A	TNFRSF10A
Tumor necrosis factor receptor superfamily member 11A	TNFRSF11A
Tumor necrosis factor receptor superfamily member 13B	TNFRSF13B
Tyrosine-protein kinase Mer	MERTK
V-set and immunoglobulin domain-containing protein 2	VSIG2
Vascular endothelial growth factor D	VEGFD

S.3.

Demographics and characteristics of patients with first episode psychosis stratified by patient group and healthy controls.

	Median [IQR] (nr)				
**Characteristic**	**HC (54)**	**SCZ (42)**	**Non-SCZ (14)**	***p*-value HC vs SCZ**	***p*-value HC vs non-SCZ**
**Gender, male/female**	26/28 (54)	27/15 (42)	8-6 (14)	0.12 [a]	0.55 [a]
**Age, years**	26 [23-30] (54)	29.0 [24.5-34] (42)	27.0 [24.3-32.0] (14)	0.10 [b]	0.35 [b]
**BMI, kg/m $^{-2}$**	23.0 [21.0-26.0] (51)	23.0 [21.0-25.2] (42)	23.0 [21.8-26.3] (14)	0.23 [b]	0.96 [b]
**Nicotine use, %**	13 (53)	20 (41)	46 (13)	0.41 [a]	0.01 [a]
**Smoking, %**	4 (53)	10 (42)	0 (14)	0.24 [a]	0.47 [a]
**DUP, months**	-	6 [2-18] (41)	3.0 [1-5] (13)	-	-
**Under medication, nr**					
**Antipsychotics**	0 (54)	26 (42)	2 (14)	-	-
**Antidepres-sants**	0 (54)	5 (42)	1 (14)	-	-
**Benzodiaze-pines/ Benzodiaze-pine-like drugs**	0 (54)	20 (42)	5 (14)	-	-
**Phenothiazine derivates**	0 (54)	9 (42)	2 (14)	-	-
**Antimanic agents**	0 (54)	1 (42)	0 (14)	-	-
**PANSS**					
**Positive**	-	19 [13-24] (42)	16 [13-21] (14)	-	-
**Negative**	-	17 [10-22] (42)	16 [12-21] (14)	-	-
**General**	-	35 [31-44] (42)	41 [27-47] (14)	-	-
**Total**	-	73 [61-84] (42)	72 [54-91] (14)	-	-
**Level of functioning**	-				
**CGI**	-	5 [4-5] (42)	4 [3-5] (14)	-	-
**GAF symptoms**	-	31 [26-36] (42)	32 [29-40] (14)	-	-
**GAF function**	-	40 [34-46] (42)	43 [34-57] (42)	-	-

The patients were stratified based on diagnosis of schizophrenia or schizoaffective disorder (SCZ) or no such diagnosis (non-SCZ) after 1,5-year follow-up. Each patient group were compared separately with the healthy control group.

Abbreviations: BMI = Body Mass Index, DUP = duration of untreated psychosis, PANSS = Positive and Negative Syndrome Scale, CGI= Clinical Global Impression and GAF= Global Assessment of Functioning.

a: Chi square test

b: Mann Whitney U test

S.4.

OLINK proteomics in plasma of patients with first episode psychosis vs. healthy controls.

**Biomarker**	**Ave Expr FEP (NPX)**	**Ave Expr HC (NPX)**	**Log_2FC (NPX)**	**Adj. *p*-value**	**Detectable samples (%)**	**Detectable samples FEP (%)**	**Detectable samples HC (%)**
**NEMO**	5.98	4.55	1.600	1.36E-08	100%	100%	100%
**ITGB1BP2**	3.30	2.09	1.369	2.46E-08	34%	49%	20%
**GLO1**	6.51	5.67	0.928	3.27E-08	100%	100%	100%
**DECR1**	4.56	3.20	1.540	4.71E-08	72%	81%	60%
**STK4**	3.52	2.17	1.494	5.41E-08	95%	99%	89%
**SRC**	5.80	4.45	1.508	3.20E-07	100%	100%	100%
**HSP 27**	9.34	8.56	0.887	3.84E-06	100%	100%	100%
**CD40-L**	3.19	2.40	0.958	1.32E-05	98%	99%	95%
**CCL17**	7.56	6.90	0.737	3.33E-05	100%	100%	100%
**CXCL1**	9.31	8.58	0.831	0.00038	100%	100%	100%
**LOX-1**	5.80	5.43	0.414	0.00105	100%	100%	100%
**Dkk-1**	7.28	6.94	0.393	0.00112	100%	100%	100%
**THBS2**	5.28	5.40	-0.117	0.00218	100%	100%	100%
**PAR-1**	7.69	7.45	0.340	0.00346	100%	100%	100%
**PDGF subu-nit B**	8.01	7.50	0.603	0.00574	100%	100%	100%
**CEACAM8**	2.85	2.63	0.300	0.00606	98%	96%	100%
**HAOX1**	4.47	3.81	0.731	0.01286	100%	100%	100%
**CA5A**	1.60	1.18	0.458	0.01663	33%	36%	29%
**ANGPT1**	7.27	6.90	0.448	0.01832	100%	100%	100%

**IL1RL2**	3.51	3.30	0.233	0.02137	100%	100%	100%
**CD84**	3.96	3.83	0.208	0.02774	100%	100%	100%
**HB-EGF**	4.15	4.02	0.228	0.03706	100%	100%	100%
**IL-1ra**	3.97	3.78	0.237	0.03778	100%	100%	100%
**BNP**	0.08	0.31	-0.185	0.04122	11%	8%	15%
**SPON2**	7.91	8.02	-0.105	0.04842	100%	100%	100%
**GDF-2**	7.67	7.97	-0.224	0.04905	100%	100%	100%
**PARP-1**	2.44	2.23	0.261	0.05126	91%	88%	95%
**GT**	1.56	1.79	-0.208	0.05353	93%	89%	98%
**IDUA**	5.02	4.86	0.195	0.06241	100%	100%	100%
**CD4**	3.86	4.02	-0.118	0.07439	100%	100%	100%
**ADAM-TS13**	4.82	4.90	-0.057	0.07619	100%	100%	100%
**RAGE**	12.70	12.88	-0.155	0.07993	100%	100%	100%
**FS**	10.00	10.21	-0.187	0.08748	100%	100%	100%
**FABP2**	7.34	7.67	-0.291	0.09525	100%	100%	100%
**TGM2**	6.22	6.07	0.197	0.09603	100%	100%	100%
**IL-17D**	1.24	1.39	-0.089	0.11578	52%	42%	64%
**HO-1**	10.43	10.63	-0.149	0.12865	100%	100%	100%
**ADM**	7.13	7.32	-0.155	0.12886	100%	100%	100%
**CTRC**	9.91	9.78	0.177	0.14769	100%	100%	100%
**DCN**	3.76	3.86	-0.080	0.14805	100%	100%	100%
**SOD2**	9.82	9.77	0.037	0.16227	100%	100%	100%
**PAPPA**	2.01	1.96	0.142	0.21282	89%	86%	93%
**IL-4RA**	1.22	1.19	0.073	0.21662	88%	86%	89%
**PRSS8**	7.96	7.88	0.108	0.23845	100%	100%	100%
**REN**	5.64	5.54	0.129	0.24148	100%	100%	100%

**TNFRSF13B**	8.65	8.80	-0.095	0.24841	100%	100%	100%
**IL-27**	5.55	5.67	-0.079	0.28893	100%	100%	100%
**FGF-23**	1.76	1.91	-0.093	0.30732	88%	85%	91%
**PTX3**	2.61	2.75	-0.082	0.34855	99%	99%	100%
**IL16**	5.46	5.42	0.114	0.36367	100%	100%	100%
**IL18**	7.37	7.34	0.098	0.38645	100%	100%	100%
**Gal-9**	7.25	7.32	-0.058	0.39287	100%	100%	100%
**GH**	7.55	7.98	-0.381	0.41807	100%	100%	100%
**PGF**	6.29	6.40	-0.064	0.42651	100%	100%	100%
**SORT1**	7.48	7.46	0.061	0.43056	100%	100%	100%
**XCL1**	4.60	4.56	0.102	0.43571	100%	100%	100%
**IL6**	1.65	1.60	0.087	0.44790	82%	84%	80%
**PRELP**	7.62	7.68	-0.029	0.46093	100%	100%	100%
**PRSS27**	8.02	8.13	-0.070	0.46375	100%	100%	100%
**TNFRSF11A**	4.55	4.64	-0.062	0.46446	100%	100%	100%
**ACE2**	2.40	2.37	0.076	0.47129	96%	97%	95%
**MMP12**	6.12	6.06	0.089	0.51136	100%	100%	100%
**LEP**	4.99	5.14	-0.163	0.51588	97%	99%	95%
**AMBP**	7.11	7.10	0.029	0.52017	100%	100%	100%
**THPO**	2.64	2.65	0.057	0.52061	100%	100%	100%
**KIM1**	6.77	6.72	0.069	0.54285	100%	100%	100%
**PD-L2**	2.30	2.31	0.043	0.55843	100%	100%	100%
**PIgR**	5.06	5.05	0.016	0.57637	100%	100%	100%
**VSIG2**	2.70	2.68	0.049	0.58113	99%	100%	98%
**MERTK**	5.04	5.16	-0.050	0.61387	100%	100%	100%
**MMP7**	9.03	9.02	0.048	0.62911	100%	100%	100%
**SCF**	8.71	8.76	-0.042	0.65228	100%	100%	100%

**CCL3**	4.91	4.88	0.075	0.67459	100%	100%	100%
**SLAMF7**	2.04	2.13	-0.041	0.70926	69%	66%	73%
**GIF**	6.00	5.97	0.054	0.72225	100%	100%	100%
**hOSCAR**	9.79	9.85	-0.021	0.72439	100%	100%	100%
**CTSL1**	6.37	6.38	0.022	0.72488	100%	100%	100%
**FGF-21**	4.68	4.81	-0.100	0.73424	98%	97%	98%
**PSGL-1**	3.95	3.97	0.018	0.73886	100%	100%	100%
**TIE2**	6.83	6.84	0.022	0.74258	100%	100%	100%
**SERPINA12**	2.77	2.87	-0.080	0.74379	94%	97%	89%
**TF**	4.95	5.02	-0.021	0.76675	100%	100%	100%
**MARCO**	5.67	5.70	0.010	0.80487	100%	100%	100%
**LPL**	9.82	9.87	-0.021	0.80925	100%	100%	100%
**TNFRSF10A**	1.76	1.82	-0.012	0.85982	86%	86%	85%
**IgG Fc receptor II-b**	1.88	1.98	-0.021	0.89131	82%	82%	82%
**VEGFD**	7.28	7.32	-0.012	0.89523	100%	100%	100%
**TRAIL-R2**	4.84	4.91	-0.010	0.90337	100%	100%	100%
**BOC**	3.24	3.28	-0.006	0.94106	100%	100%	100%
**AGRP**	4.49	4.54	-0.005	0.96379	100%	100%	100%
**BMP-6**	3.56	3.60	-0.003	0.96711	100%	100%	100%
**TM**	8.48	8.53	0.000	0.99949	100%	100%	100%

For each biomarker, average expression of FEP patients and HC, the $\log_2$ fold change between the groups and the *p*-value adjusted for plate and multiple comparisons are presented. Adjustment for multiple comparisons was performed using Benjamini-Hochberg correction method and was corrected for 92 tests. The unit is the Log2- scaled NPX, where high NPX values equals a high protein concentration and 1 NPX difference means a doubling of protein concentration. Detectable samples are the percentage of samples over LOD for each biomarker.

Abbreviations: Ave Expr= Average expression, FEP = first episode psychosis, HC = healthy controls, NPX = Normalized Protein eXpression, log_2FC = log_2 fold change, Adj. *p*-value= *p*-value adjusted for plate and multiple comparisons.

S.5.

Difference in average expression of antipsychotic medicated vs non-medicated patients with first episode psychosis.

**Biomarker**	**Log_2FC FEP with antipsychotics-FEP without antipsychotics (NPX)**	**t**	**Adj. *p*-value[1]**	**Adj. *p*-value[2]**
**KIM1**	0,34	2,29	0,02	0,97
**AGRP**	0,33	2,24	0,03	0,97
**PARP-1**	0,33	2,06	0,04	0,97
**IL-1ra**	0,29	1,94	0,05	0,97
**CEACAM8**	0,23	1,76	0,08	0,97
**ACE2**	0,24	1,75	0,08	0,97
**Gal-9**	0,16	1,74	0,08	0,97
**PIgR**	0,06	1,66	0,10	0,97
**XCL1**	0,28	1,59	0,11	0,97
**IL6**	0,23	1,59	0,11	0,97
**HB-EGF**	-0,21	-1,42	0,16	0,97
**SPON2**	0,10	1,39	0,17	0,97
**PSGL-1**	0,09	1,29	0,20	0,97
**IL-4RA**	0,09	1,24	0,22	0,97
**AMBP**	0,07	1,20	0,23	0,97
**MMP12**	0,20	1,09	0,28	0,97
**PTX3**	0,12	1,02	0,31	0,97
**SRC**	0,38	1,00	0,32	0,97
**TGM2**	0,16	0,99	0,32	0,97

**IgG Fc receptor II-b**	0,17	0,93	0,35	0,97
**CA5A**	0,19	0,47	0,64	0,97
**LEP**	0,28	0,92	0,36	0,97
**BMP-6**	-0,08	-0,92	0,36	0,97
**FABP2**	-0,21	-0,92	0,36	0,97
**HSP 27**	0,23	0,91	0,36	0,97
**PRSS8**	0,11	0,91	0,36	0,97
**LPL**	0,10	0,86	0,39	0,97
**THBS2**	-0,04	-0,86	0,39	0,97
**CCL17**	0,19	0,84	0,40	0,97
**GIF**	-0,17	-0,83	0,41	0,97
**SLAMF7**	-0,09	-0,66	0,51	0,97
**MMP7**	-0,11	-0,79	0,43	0,97
**GDF-2**	-0,11	-0,76	0,45	0,97
**THPO**	0,09	0,76	0,45	0,97
**ITGB1BP2**	-0,09	-0,33	0,74	0,97
**TRAIL-R2**	0,08	0,75	0,46	0,97
**NEMO**	0,26	0,73	0,47	0,97
**GLO1**	0,15	0,71	0,48	0,97
**STK4**	0,24	0,69	0,49	0,97
**TNFRSF11A**	0,08	0,70	0,49	0,97
**IL-17D**	-0,01	-0,21	0,83	0,97
**GH**	0,40	0,64	0,52	0,97
**SOD2**	-0,02	-0,64	0,52	0,97
**FGF-21**	-0,21	-0,56	0,58	0,97
**PGF**	0,06	0,55	0,58	0,97

**CD4**	0,05	0,54	0,59	0,97
**ADM**	0,07	0,52	0,60	0,97
**REN**	-0,08	-0,52	0,60	0,97
**TM**	0,06	0,49	0,62	0,97
**PAPPA**	0,05	0,36	0,72	0,97
**PRELP**	-0,02	-0,46	0,65	0,97
**FGF-23**	-0,04	-0,33	0,74	0,97
**HO-1**	-0,06	-0,45	0,66	0,97
**TNFRSF13B**	0,05	0,44	0,66	0,97
**LOX-1**	0,07	0,44	0,66	0,97
**VEGFD**	0,05	0,43	0,66	0,97
**TF**	-0,04	-0,42	0,68	0,97
**HAOX1**	0,16	0,41	0,68	0,97
**DECR1**	-0,03	-0,07	0,94	0,98
**IL-27**	-0,04	-0,40	0,69	0,97
**GT**	-0,04	-0,26	0,79	0,97
**IDUA**	0,05	0,38	0,71	0,97
**CTSL1**	0,03	0,38	0,71	0,97
**IL1RL2**	0,05	0,36	0,72	0,97
**FS**	0,05	0,36	0,72	0,97
**MARCO**	-0,02	-0,34	0,73	0,97
**TIE2**	-0,03	-0,32	0,75	0,97
**TNFRSF10A**	0,00	-0,03	0,98	0,98
**CD40-L**	-0,08	-0,28	0,78	0,97
**SERPINA12**	0,07	0,22	0,83	0,97
**ANGPT1**	-0,07	-0,26	0,79	0,97
**DCN**	-0,02	-0,26	0,80	0,97

**SCF**	-0,03	-0,25	0,80	0,97
**IL18**	0,04	0,25	0,81	0,97
**MERTK**	-0,03	-0,24	0,81	0,97
**VSIG2**	-0,03	-0,24	0,81	0,97
**PAR-1**	-0,04	-0,24	0,81	0,97
**RAGE**	0,03	0,23	0,82	0,97
**CCL3**	-0,05	-0,22	0,83	0,97
**SORT1**	-0,02	-0,21	0,83	0,97
**ADAM-TS13**	-0,01	-0,20	0,84	0,97
**hOSCAR**	0,01	0,18	0,86	0,97
**CTRC**	-0,03	-0,16	0,87	0,98
**PD-L2**	-0,01	-0,13	0,90	0,98
**PRSS27**	-0,02	-0,13	0,90	0,98
**IL16**	-0,02	-0,11	0,91	0,98
**PDGF subunit B**	-0,02	-0,08	0,94	0,98
**Dkk-1**	-0,01	-0,07	0,95	0,98
**CXCL1**	-0,02	-0,06	0,95	0,98
**CD84**	-0,01	-0,04	0,97	0,98
**BOC**	0,00	-0,03	0,97	0,98

P-values are adjusted for plate and multiple comparisons (1) and age, gender, nicotine use, plate and multiple comparison (2). Adjustment for multiple comparisons was performed using Benjamini-Hochberg correction method and was corrected for 91 tests.

Abbreviations: FEP = first episode psychosis, NPX = Normalized Protein eXpression, log_2FC = log_2 fold change, Adj. *p*-value= adjusted *p*-value.

S.6.

OLINK proteomics in plasma of patients with first episode psychosis vs. healthy controls adjusted for plate, age, gender, nicotine use and multiple comparisons.

Biomarker	Log_2 FC FEP-HC (NPX)	Ave Expr FEP	t	*P* -value	Adj. *p*-value
NEMO	1,51	5,4	5,8	3,35E-08	3,05E-06
GLO1	0,87	6,21	5,46	1,68E-07	7,63E-06
SRC	1,42	5,3	5,05	1,15E-06	3,48E-05
STK4	1,27	3,13	4,99	1,59E-06	3,62E-05
HSP 27	0,83	9,07	4,5	1,29E-05	2,35E-04
DECR1	1,13	4,58	4,26	4,02E-05	5,72E-04
CD40-L	0,9	2,85	4,2	4,40E-05	5,72E-04
CCL17	0,71	7,23	4,07	7,17E-05	8,15E-04
CA5A	1,12	2,57	4,02	1,82E-04	1,84E-03
CXCL1	0,73	8,92	3,21	1,61E-03	1,36E-02
CEACAM8	0,33	2,86	3,2	1,65E-03	1,36E-02
LOX-1	0,38	5,68	3,1	2,26E-03	1,72E-02
THBS2	-0,11	5,33	-3	3,13E-03	2,19E-02
Dkk-1	0,35	7,09	2,93	3,91E-03	2,54E-02
PARP-1	0,33	2,53	2,71	7,48E-03	4,54E-02
PAR-1	0,31	7,62	2,68	8,12E-03	4,62E-02
PDGF subu-nit B	0,54	7,72	2,5	1,34E-02	7,19E-02
HAOX1	0,61	4,32	2,23	2,68E-02	1,36E-01

ANGPT1	0,4	7,04	2,13	3,45E-02	1,65E-01
IL-1ra	0,21	3,94	1,98	4,93E-02	1,99E-01
IL1RL2	0,2	3,46	2,02	4,53E-02	1,99E-01
CD84	0,19	3,93	2,01	4,60E-02	1,99E-01
SPON2	-0,1	7,98	-1,97	5,04E-02	1,99E-01
ADAM-TS13	-0,06	4,89	-1,87	6,38E-02	2,15E-01
CD4	-0,13	3,98	-1,9	5,87E-02	2,15E-01
RAGE	-0,16	12,82	-1,85	6,65E-02	2,15E-01
ADM	-0,18	7,29	-1,83	6,85E-02	2,15E-01
HO-1	-0,19	10,59	-1,87	6,28E-02	2,15E-01
FS	-0,2	10,17	-1,84	6,81E-02	2,15E-01
CTRC	0,21	9,88	1,74	8,44E-02	2,27E-01
HB-EGF	0,19	4,12	1,77	7,82E-02	2,27E-01
IDUA	0,19	4,99	1,75	8,26E-02	2,27E-01
DCN	-0,1	3,84	-1,77	7,93E-02	2,27E-01
GDF-2	-0,2	7,91	-1,73	8,46E-02	2,27E-01
TGM2	0,19	6,25	1,57	1,19E-01	3,09E-01
ITGB1BP2	0,37	4,47	1,54	1,30E-01	3,28E-01
FABP2	-0,26	7,54	-1,45	1,48E-01	3,64E-01
IL-4RA	0,07	1,35	1,37	1,72E-01	4,11E-01
PAPPA	0,14	2,24	1,35	1,79E-01	4,17E-01
PGF	-0,1	6,4	-1,22	2,25E-01	5,11E-01
IL-17D	-0,05	1,66	-1,17	2,43E-01	5,39E-01
LEP	-0,21	5,22	-1,09	2,79E-01	6,04E-01
SOD2	0,03	9,78	0,97	3,33E-01	6,50E-01
PRELP	-0,04	7,7	-0,98	3,27E-01	6,50E-01

TNFRSF13B	-0,08	8,78	-0,98	3,29E-01	6,50E-01
PRSS27	-0,09	8,09	-0,99	3,24E-01	6,50E-01
MERTK	-0,09	5,21	-0,97	3,36E-01	6,50E-01
IL-27	-0,07	5,65	-0,95	3,44E-01	6,52E-01
XCL1	0,11	4,64	0,86	3,93E-01	7,06E-01
Gal-9	-0,06	7,29	-0,85	3,95E-01	7,06E-01
TNFRSF11A	-0,07	4,63	-0,87	3,85E-01	7,06E-01
PRSS8	0,07	7,96	0,8	4,27E-01	7,48E-01
SERPINA12	-0,18	2,94	-0,76	4,49E-01	7,70E-01
CCL3	0,11	4,99	0,59	5,58E-01	7,89E-01
MMP12	0,08	6,1	0,62	5,33E-01	7,89E-01
IL16	0,07	5,56	0,58	5,59E-01	7,89E-01
KIM1	0,07	6,79	0,63	5,28E-01	7,89E-01
REN	0,07	5,61	0,64	5,20E-01	7,89E-01
SORT1	0,05	7,49	0,61	5,41E-01	7,89E-01
TNFRSF10A	-0,04	1,98	-0,7	4,83E-01	7,89E-01
TF	-0,04	5,04	-0,58	5,64E-01	7,89E-01
TM	-0,05	8,57	-0,58	5,59E-01	7,89E-01
PTX3	-0,06	2,72	-0,65	5,14E-01	7,89E-01
GT	-0,07	1,78	-0,69	4,92E-01	7,89E-01
FGF-21	-0,18	4,94	-0,66	5,09E-01	7,89E-01
MMP7	0,05	9,1	0,54	5,87E-01	7,98E-01
hOSCAR	-0,03	9,87	-0,55	5,82E-01	7,98E-01
IL18	0,05	7,46	0,47	6,36E-01	8,27E-01
THPO	0,04	2,72	0,47	6,36E-01	8,27E-01
SCF	-0,05	8,76	-0,5	6,19E-01	8,27E-01

SLAMF7	0,03	2,43	0,36	7,17E-01	9,14E-01
TRAIL-R2	-0,03	4,95	-0,35	7,24E-01	9,14E-01
VSIG2	0,03	2,75	0,3	7,62E-01	9,25E-01
BMP-6	-0,02	3,6	-0,32	7,53E-01	9,25E-01
FGF-23	-0,03	2	-0,31	7,56E-01	9,25E-01
IgG Fc receptor II-b	-0,04	2,28	-0,29	7,74E-01	9,27E-01
BOC	-0,02	3,3	-0,27	7,89E-01	9,33E-01
PD-L2	0,02	2,38	0,25	8,05E-01	9,39E-01
ACE2	-0,02	2,49	-0,22	8,23E-01	9,48E-01
AMBP	0,01	7,12	0,17	8,61E-01	9,56E-01
PIgR	0,01	5,08	0,19	8,53E-01	9,56E-01
VEGFD	-0,02	7,34	-0,19	8,51E-01	9,56E-01
IL6	0,01	1,88	0,12	9,02E-01	9,84E-01
AGRP	0,01	4,55	0,07	9,45E-01	9,84E-01
LPL	0,01	9,86	0,07	9,47E-01	9,84E-01
PSGL-1	0,01	4,02	0,1	9,19E-01	9,84E-01
CTSL1	0	6,44	0,06	9,52E-01	9,84E-01
MARCO	0	5,75	0,08	9,36E-01	9,84E-01
TIE2	0	6,88	-0,04	9,71E-01	9,93E-01
GIF	0	5,99	0	9,96E-01	9,96E-01
GH	0	7,71	-0,01	9,91E-01	9,96E-01

For each biomarker, average expression of FEP patients, the log_2 fold change between FEP patients and HC and *p*-value and *p*-value adjusted for plate, age, gender, nicotine use and multiple comparisons are presented. Adjustment for multiple comparisons was performed using Benjamini-Hochberg correction method

and was corrected for 91 tests. The unit is the Log2- scaled NPX, where high NPX values equals a high protein concentration and 1 NPX difference means a doubling of protein concentration.

Abbreviations: Ave Expr= Average expression, FEP = first episode psychosis, HC = healthy controls, NPX = Normalized Protein eXpression, log_2FC = log_2 fold change, Adj. *p*-value= *p*-value adjusted for plate, age, gender, nicotine use and multiple comparisons.

S.7

Difference in cardiovascular protein profiling in plasma between patients with first episode psychosis later diagnosed with schizophrenia or schizoaffective disorder and healthy controls.

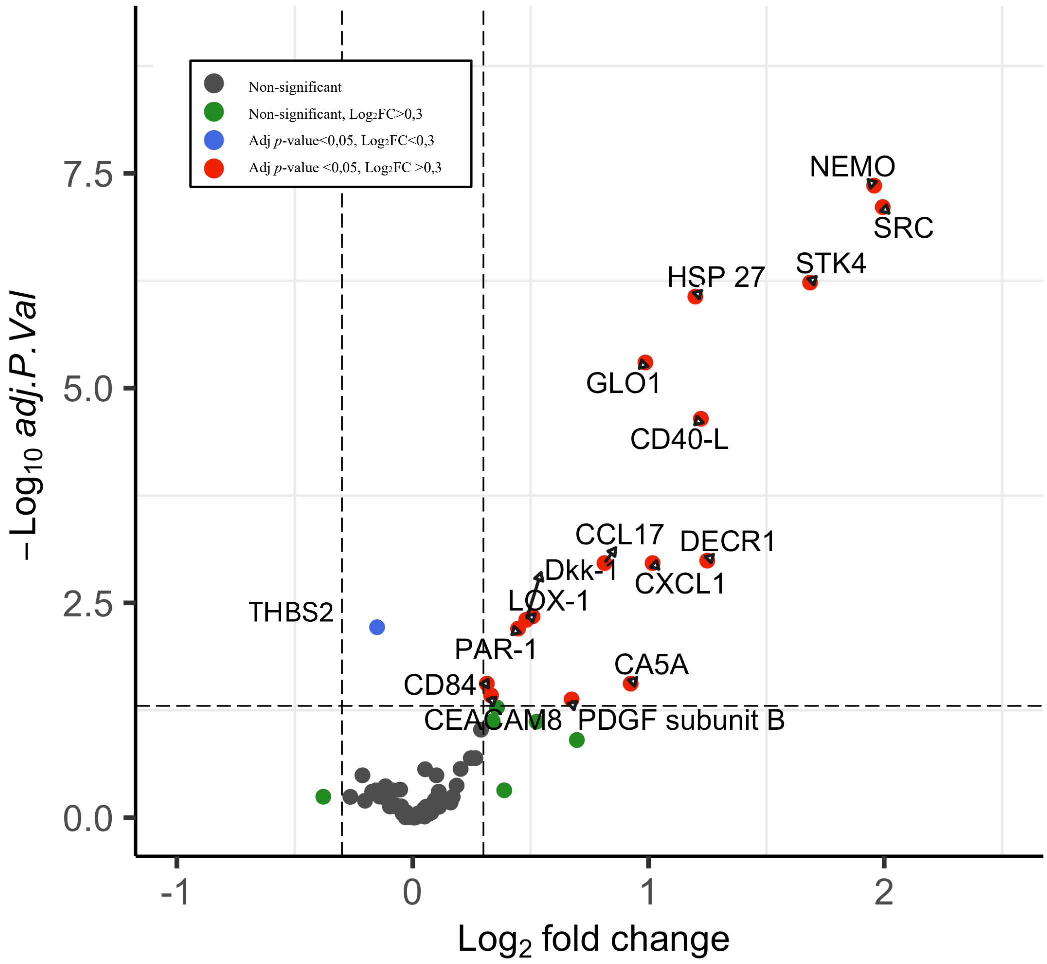

91 biomarkers are presented in the volcano plot with $\log_2$ fold change between SCZ patients and HC. The unit of the $\log_2$ fold change is the Log2- scaled NPX, where high NPX values equals a high protein concentration and 1 NPX difference means a doubling of protein concentration. Log_2 fold changes between groups and *p* values are calculated with limma package in R and adjusted for plate, age, gender and nicotine use. Adjustment for multiple comparisons was performed using Benjamini-Hochberg correction method and was corrected for 91 tests. Significance level chosen for adjusted *p* value is < 0.05.

S.8.

OLINK proteomics in plasma of patients with first episode psychosis later diagnosed with schizophrenia or schizoaffective disorder vs. healthy controls adjusted for plate, age, gender, nicotine use and multiple comparisons.

Biomarker	logFC	AveExpr SCZ	t	*P*-value	Adj. *p*-value
NEMO	1,96	5,40	6,63	4,82E-10	4,38E-08
SRC	1,99	5,30	6,39	1,71E-09	7,80E-08
STK4	1,69	3,13	5,92	1,94E-08	5,90E-07
HSP 27	1,20	9,07	5,78	3,77E-08	8,58E-07
GLO1	0,99	6,21	5,36	2,76E-07	5,02E-06
CD40-L	1,22	2,85	5,00	1,50E-06	2,27E-05
DECR1	1,25	4,58	4,09	7,83E-05	1,02E-03
CCL17	0,81	7,23	3,98	1,02E-04	1,09E-03
CXCL1	1,02	8,92	3,97	1,08E-04	1,09E-03
LOX-1	0,51	5,68	3,55	5,00E-04	4,55E-03
Dkk-1	0,48	7,09	3,50	6,01E-04	4,97E-03
THBS2	-0,15	5,33	-3,42	8,01E-04	6,07E-03
PAR-1	0,45	7,62	3,38	9,00E-04	6,30E-03
CA5A	0,92	2,57	2,98	4,38E-03	0,03
CD84	0,31	3,93	2,88	4,54E-03	0,03
CEACAM8	0,33	2,86	2,75	6,65E-03	0,04
PDGF subunit B	0,67	7,72	2,69	7,86E-03	0,04
CTRC	0,36	9,88	2,59	0,01	0,05
PARP-1	0,34	2,53	2,44	0,02	0,08

ANGPT1	0,53	7,04	2,42	0,02	0,08
HB-EGF	0,29	4,12	2,32	0,02	0,09
HAOX1	0,70	4,32	2,19	0,03	0,12
TGM2	0,27	6,25	1,95	0,05	0,20
IL-1ra	0,25	3,94	1,94	0,05	0,20
IL1RL2	0,20	3,46	1,80	0,07	0,27
SOD2	0,05	9,78	1,77	0,08	0,27
IL-4RA	0,10	1,35	1,66	0,10	0,32
FS	-0,21	10,17	-1,66	0,10	0,32
ITGB1BP2	0,39	4,47	1,38	0,17	0,48
IDUA	0,19	4,99	1,50	0,14	0,42
CD4	-0,12	3,98	-1,47	0,14	0,43
ADAM-TS13	-0,05	4,89	-1,41	0,16	0,47
SPON2	-0,08	7,98	-1,36	0,17	0,48
HO-1	-0,16	10,59	-1,35	0,18	0,48
GDF-2	-0,17	7,91	-1,30	0,19	0,50
PD-L2	0,11	2,38	1,29	0,20	0,50
ADM	-0,14	7,29	-1,20	0,23	0,57
FGF-21	-0,38	4,94	-1,18	0,24	0,57
LEP	-0,26	5,22	-1,15	0,25	0,57
IL16	0,17	5,56	1,16	0,25	0,57
THPO	0,11	2,72	1,10	0,27	0,61
VSIG2	0,11	2,75	1,06	0,29	0,62
REN	0,13	5,61	1,06	0,29	0,62
SORT1	0,09	7,49	1,04	0,30	0,62
RAGE	-0,11	12,82	-1,02	0,31	0,62
PAPPA	0,12	2,24	1,01	0,32	0,63

FABP2	-0,20	7,54	-0,98	0,33	0,64
GIF	0,16	5,99	0,93	0,35	0,67
SLAMF7	0,09	2,43	0,83	0,41	0,73
IL-27	-0,08	5,65	-0,88	0,38	0,71
BOC	0,08	3,30	0,85	0,40	0,72
GT	-0,10	1,78	-0,80	0,42	0,74
IL-17D	-0,03	1,66	-0,53	0,60	0,86
PGF	-0,07	6,40	-0,77	0,44	0,74
KIM1	0,10	6,79	0,77	0,44	0,74
Gal-9	-0,06	7,29	-0,76	0,45	0,74
DCN	-0,05	3,84	-0,75	0,46	0,74
XCL1	0,11	4,64	0,72	0,47	0,75
BMP-6	0,06	3,60	0,71	0,48	0,75
IL6	0,08	1,88	0,63	0,53	0,79
IgG Fc receptor II-b	0,07	2,28	0,42	0,67	0,90
AGRP	0,08	4,55	0,62	0,53	0,79
TIE2	0,05	6,88	0,62	0,53	0,79
IL18	0,08	7,46	0,62	0,54	0,79
MMP12	0,08	6,10	0,51	0,61	0,87
LPL	0,05	9,86	0,50	0,62	0,87
PRSS8	0,05	7,96	0,46	0,65	0,89
VEGFD	0,04	7,34	0,43	0,67	0,90
AMBP	0,02	7,12	0,41	0,68	0,90
MMP7	0,04	9,10	0,39	0,70	0,90
TNFRSF13B	-0,04	8,78	-0,38	0,70	0,90
MERTK	-0,04	5,21	-0,37	0,71	0,90

ACE2	0,03	2,49	0,29	0,77	0,94
PTX3	-0,03	2,72	-0,31	0,76	0,94
TNFRSF11A	-0,03	4,63	-0,30	0,76	0,94
TNFRSF10A	0,01	1,98	0,20	0,84	0,97
SCF	0,03	8,76	0,26	0,80	0,96
TM	0,02	8,57	0,24	0,81	0,96
SERPINA12	0,05	2,94	0,18	0,85	0,97
PRELP	-0,01	7,70	-0,20	0,84	0,97
FGF-23	0,01	2,00	0,05	0,96	0,99
CTSL1	0,01	6,44	0,14	0,89	0,99
TRAIL-R2	-0,01	4,95	-0,13	0,90	0,99
TF	0,01	5,04	0,10	0,92	0,99
MARCO	0,00	5,75	0,10	0,92	0,99
GH	-0,03	7,71	-0,07	0,95	0,99
CCL3	0,01	4,99	0,06	0,96	0,99
PSGL-1	0,00	4,02	0,04	0,97	0,99
PRSS27	0,00	8,09	-0,02	0,98	0,99
PIgR	0,00	5,08	0,02	0,99	0,99
hOSCAR	0,00	9,87	0,01	0,99	0,99

For each biomarker, average expression of SCZ patients, the log_2 fold change between SCZ patients and HC and *p*-value adjusted for plate, age, gender, nicotine use and multiple comparisons are presented. Adjustment for multiple comparisons was performed using Benjamini-Hochberg correction method and was corrected for 91 tests. The unit is the Log2- scaled NPX, where high NPX values equals a high protein concentration and 1 NPX difference means a doubling of protein concentration.

Abbreviations: Ave Expr= Average expression, SCZ =patient with first episode psychosis, later diagnosed with schizophrenia/schizoaffective disorder, NPX = Normalized Protein eXpression, log_2FC = log_2 fold change, Adj. *p*-value= *p*-value adjusted for plate, age, gender, nicotine use and multiple comparisons.

S.9

Correlations of significant different biomarkers in plasma (NPX) and cardiovascular risk factors in patients with first episode psychosis.

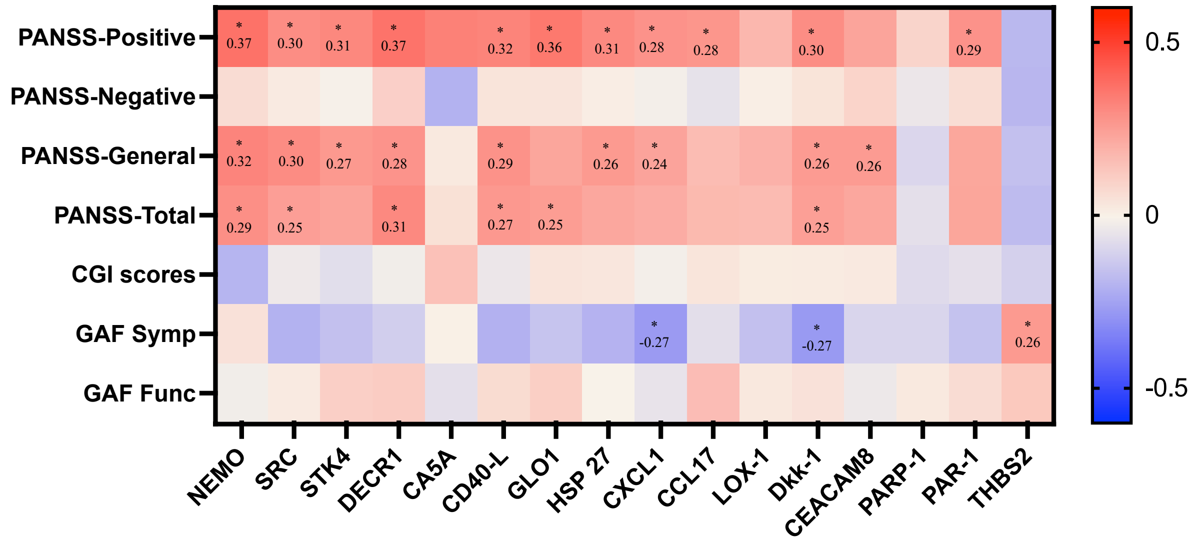

Correlation analysis was performed using Spearman correlation (r_s) and numbers in the heat map boxes indicate r_s values. The color represents the value of the Spearman's rank coefficient and red is a positive correlation and blue a negative correlation. The asterix indicates a significant correlation ($p<0.05$). None of the correlations remained significant after correction for multiple analysis with Benjamini Hochberg's method.

Abbreviations: HDL= High density lipoprotein and LDL= Low density lipoprotein
